# Betaine addition to the diet alleviates intestinal injury in growing rabbits during the summer heat through the AAT/mTOR pathway

**DOI:** 10.1186/s40104-024-00998-6

**Published:** 2024-03-08

**Authors:** Zimei Li, Junning Pu, Xiang Chen, Yanbin Chen, Xiaoyan Peng, Jingyi Cai, Gang Jia, Hua Zhao, Gang Tian

**Affiliations:** Animal Nutrition Institute, Sichuan Agricultural University, Chengdu, Sichuan 611130 China

**Keywords:** AAT/mTOR, Betaine, Growing rabbits, Intestinal injury, Summer heat

## Abstract

**Background:**

The aim of this experiment was to investigate the effect of different levels of betaine (Bet) inclusion in the diet on the intestinal health of growing rabbits under summer heat. A total of 100 weaned Qixing meat rabbits aged 35 d with body weight of 748.61 ± 38.59 g were randomly divided into 5 treatment groups: control group (basal diet) and Bet groups (basal diet + 0.75, 1.0, 1.5 or 2.0 g/kg Bet). The average daily temperature in the rabbitry during the experiment was 30.48 °C and the relative humidity was 69.44%.

**Results:**

Dietary addition of Bet had no significant effect on growth performance and health status of growing rabbits (*P* > 0.05), but it increased ileal secretory immunoglobulin A content compared to the control under summer heat (*P* < 0.05). Addition of 0.75 g/kg Bet up-regulated jejunal *IL-4*, down-regulated ileal *TNF-α* expression (*P* < 0.05). The addition of 1.0 g/kg Bet increased the villi height (VH) in the jejunum (*P* < 0.05). Serum glucose levels were reduced, and the expression of *SLC6A20* was up-regulated in jejunum and ileum of rabbits fed with 1.5 g/kg Bet (*P* < 0.05). When added at 2.0 g/kg, Bet reduced serum HSP70 content, increased jejunal VH, and up-regulated duodenal *SLC7A6*, *SLC38A2*, *mTOR* and *4EBP-2* expression (*P* < 0.05). Correlation analysis revealed that intestinal *mTOR* expression was significantly and positively correlated with *SLC7A6*, *SLC38A2*, *SLC36A1* and *IL-4* expression (*P* < 0.05).

**Conclusions:**

Dietary addition of Bet can up-regulate the expression of anti-inflammatory factors through the AAT/mTOR pathway, improve the intestinal immune function, alleviate intestinal damage in growing rabbits caused by summer heat, and improve intestinal health.

## Background

As global temperatures rise, hot and humid environments are becoming one of the main problems faced by animal husbandry in many parts of the world. Sustained summer heat challenges can put animals under prolonged stress, leading to increased morbidity, decreased growth performance, impaired immune function, and serious economic losses [1–3]. As an animal lacking functional sweat glands, rabbits mainly rely on respiration and blood flow through their ears to dissipate heat. When the breeding environment is too hot in summer, it is difficult for rabbits to release excess heat from their bodies, and rabbits are prone to stress and even death [4]. As one of the most important immune organs in the animal body, the intestine is vital for the growth and development of the animal [5]. After weaning, the intestinal development of newborn rabbits is still incomplete and susceptible to high temperature, which will block further development, trigger oxidative stress, produce inflammatory response, and damage intestinal health, resulting in decreased growth performance and increased morbidity and mortality of rabbits [6–8]. Therefore, effective approaches to alleviate the adverse effects of summer heat on rabbits need to be explored.

Betaine (Bet), as a methyl donor feed additive for animals which has significant effects on promoting animal growth, relieving stress and maintaining intestinal health [9, 10]. Addition of 1.0 g/kg Bet to the broiler diets can improve intestinal morphology, reduces intestinal permeability and improves the immune function of the organism [11]. Dietary 0.1% Bet significantly increased the serum immunoglobulin content of broilers under high temperature environment [12]. Dietary supplementation of 1.0 g/kg Bet also down-regulated the expression of inflammatory factor *IL-1β* in the intestinal tract of broilers, which alleviated the inflammatory response induced by high temperature [13]. Bet as a glycine derivative can be transported by amino acid transporters (AAT), and mTOR is a central regulator responsible for integrating various cellular signals, especially those from AAT [14]. Bet is transported by amino acid transporter A2 (SNAT2) [15], which also activates the downstream signaling factors P70 ribosomal protein S6 kinase 1 (P70S6k1) and eukaryotic translation initiation factor 4E binding protein-2 (4EBP-2) to maintain intestinal health [14, 16]. SNAT2 is composed of solute carrier (SLC) gene family [17], of which SLC38A2 and SLC7A5 have been reported to regulate protein synthesis as well as cell growth and proliferation by activating the mTOR signaling pathway [18]. Dietary Bet can also reduce oxidative stress and inflammatory response through mTOR signaling pathway in Hu sheep [19]. Therefore, we speculate that Bet may enhance body immunity and maintain intestinal health in growing rabbits through AAT/mTOR pathway. However, it has not been reported that Bet can improve the intestinal injury induced by summer heat in rabbits by upregulating the gene expression of mTOR and its downstream signaling factors through SNAT2 or other AATs that transport Bet in the rabbit intestine. Thus, this study aims to investigate the effects of dietary supplementation with different levels of Bet on the intestinal health of growing rabbits under the summer heat and to reveal the potential alleviating effect of Bet on the intestinal injury of growing rabbits and its possible mechanism.

## Materials and methods

All experimental animal procedures were approved by the Animal Care and Use Committee of Sichuan Agricultural University (approval number: SICAU20220703).

### Experimental design

A single-factor experimental design was used in this study, and 100 weaned Qixing meat rabbits (German white rabbit × Sichuan white rabbit), 35 days old with body weight of 748.61 ± 38.59 g, half male and half female, were purchased from Hongzhan Family Farm (Jintang County, Chengdu City, Sichuan Province, China). Rabbits were randomly divided into 5 treatment groups, which were supplemented with 0 (control group), 0.75, 1.0, 1.5 and 2.0 g/kg Bet (Yixing Tianshi Feed Co., Ltd., Jiangsu, China) in the basal diet, respectively. Each treatment had 20 replicates and one rabbit in each replicate. The animals were kept in single cages (galvanised wire cages) throughout the 21 d experiment [20].

The experimental basal diet was based on alfalfa, corn, wheat bran and soybean meal, and was formulated according to the nutritional requirements of growing and fattening rabbits [20]. The experimental diets were supplemented with 0.75, 1.0, 1.5 and 2.0 g/kg Bet in the form of equivalent replacement of rice husk powder in the basal diet. All diets were made into pellet feeds with a diameter of 2.5 mm and a length of 6 to 12 mm, packaged in moisture-proof feed bags, and stored in a dry and ventilated place before use. Basal diet composition and nutrient levels are shown in Table 1.

**Table 1:** Composition and nutrient levels of diets (dry matter basis, %)

**Ingredients**	**Inclusion, %**	**Nutrient**	**Level**^**2**^
Alfalfa meal	20.00	DE, MJ/kg	10.20 (10.39)
Corn	13.10	CP	16.10 (15.90)
Soybean meal	10.90	CF	16.00 (16.23)
Corn germ meal	10.00	NDF	33.20 (44.50)
Wheat bran	20.00	ADF	18.51 (21.68)
Soybean hull	12.25	ADL	3.66 (4.15)
Rice bran	4.42	Ca	0.60
Rice hull power	5.82	TP	0.52
Soybean oil	1.31	SAA	0.52
CaCO_3_	0.55	AFD Lys	0.52
NaCl	0.50	AFD Met	0.21
L-Lys HCl (≥ 98%)	0.03	AFD Thr	0.43
L-Met (≥ 99%)	0.03		
L-Thr (≥ 98.5%)	0.09		
Premix^1^	1.00		
Total	100.00		

^1^Premix provided the following per kg of the diet: vitamin premix 250 mg (include 6,000 IU of VA, 1,200 IU of VD_3_, 50 IU of VE, 2.4 mg of VK_3_, 12.5 μg of VB_12_, 240 μg of biotin, 1.8 mg of pyridoxine, 3.6 mg of riboflavin, 35 mg of niacin, 12.5 mg of pantothenic acid), 35 mg Zn (ZnSO_4_·H_2_O), 30 mg Fe (FeSO_4_·7H_2_O), 8 mg Mn (MnSO_4_·H_2_O), 6 mg Cu (CuSO_4_·5H_2_O), 0.4 mg I (KI), 0.05 mg Se (Na_2_SeO_3_), 0.3 mg Co (CoCl_2_·6H_2_O), 100 mg Choline (choline chloride, 50%)

^2^Nutrient levels are measured values in parentheses, and the rest are calculated values

The experiments were carried out in July and August, and the enclosures were thoroughly disinfected before the start of the experiment. The rabbits were then randomly allocated according to treatment to galvanised wire mesh cages (50 cm × 50 cm × 40 cm, length × width × height) with water troughs and nipple drinkers. Temperature and humidity meter were hung on the walls around the rabbitry (at a height of 1.5 m above the floor) during the experiment to record the temperature and humidity at 06:00 and 14:00 each day [21]. The rabbits were fed twice a day (08:00 and 20:00) to allow ad libitum access to food and water. The rabbitry was cleaned daily to keep it ventilated and hygienic and a detailed record was kept of the health status of the rabbits.

### Sample collection

At 06:00 h on the 22^nd^ day of the experiment, 6 rabbits (3 males and 3 females), which were close to the average body weight of the group, were selected from each treatment group. Approximately 5 mL of blood was collected from the heart with butterfly blood sampling needle and injected into anticoagulant-free blood collection tubes. After standing for 1 h at room temperature, the blood was centrifuged at 3,000 × *g* for 15 min and the supernatant was divided into centrifuge tubes and stored at −20 °C until further analysis.

After blood collection, the rabbits were electricity stunned (50 V, pulsed direct current, 60 Hz for 5 s) and killed by cervical dislocation. The duodenum, jejunum, and ileum were separated by dissecting rabbits, and 2–3 cm of tissue samples from the middle of the jejunal and ileal segments were collected. The intestinal samples were rinsed with precooled 0.9% saline and fixed in 4% paraformaldehyde fixative at room temperature in preparation of histological analysis. The jejunum was then gently squeezed with forceps and the extruded mucus was divided into lyophilized tubes and snap frozen in liquid nitrogen and stored at −80 °C in the refrigerator for testing. The remaining intestinal tissues were cut longitudinally, rinsed with pre-cooled 0.9% saline, blotted on filter paper, collected near the middle of each intestinal segment, packed in frozen tubes, snap frozen in liquid nitrogen and stored at −80 °C in the refrigerator before testing.

### Indicator measurement and analysis

#### Temperature and humidity index

The temperature and humidity of the rabbitry were used to calculate temperature and humidity index (THI) of the rabbitry according to the following formula [22]:

$${\text{THI}}\hspace{0.17em}=\hspace{0.17em}T-[(0.31-0.31{\text{RH}})\hspace{0.17em}\times \hspace{0.17em}(T-14.4)]$$

*T* refers to temperature (°C), RH refers to relative humidity (%). When THI < 27.8 (no heat stress), THI = 27.8 to 28.9 (moderate heat stress), THI = 28.9 to 30.0 (severe heat stress), THI > 30.0 (very severe heat stress).

#### Growth performance

The feed intake of rabbits was recorded daily during the experiment, and on d 1 and 21 of the experiment, all rabbits were fasting weighed and body weight (BW) was recorded. Average daily gain (ADG) and average daily feed intake (ADFI) were calculated for rabbits in each treatment group for d 1–21, and then the feed to gain ratio (F/G) was calculated [23].

#### Health status

The health status (mental state, feeding and diarrhoea, etc.) of the rabbits was monitored daily during the experiment, and their morbidity, mortality and health risk index were calculated [23]:

$$\begin{array}{lll}\text{Morbidity}\,(\%)=\text{(number of rabbits with disease in the experiment/total number of rabbits at the start of the experiment)}\times100\%;\\\text{Mortality rate}\,(\%)=\text{(number of rabbits that died in the experiment/total number of rabbits at the beginning of the experiment)}\times100\%;\\\text{Health risk index}\, (\%)=\text{(the sum of the number of rabbits that became ill and the number of rabbits that died in the experiment/the total number of rabbits at the start of the experiment)}\times100\%.\end{array}$$

When calculating the morbidity, mortality, and health risk index for each group of rabbits during the experimental period, the morbidity or death of the rabbits was counted only once.

#### Intestinal morphology

The jejunal and ileal tissue fixed in 4% paraformaldehyde were dehydrated, trimmed, dipped in wax, embedded and sectioned, and then stained with hematoxylin-eosin (HE). Samples were observed with a light microscope (40× imaging), and a randomly selected morphologically intact target area was photographed and the villi height (VH) and corresponding crypt depth (CD) were determined to calculate the villi to crypt ratio (V/C).

### Indicators of intestinal immunity

Tissue homogenates were prepared by accurately weighing 0.1 g of duodenal, jejunal and ileal tissue samples with saline at a mass-to-body ratio of 1:9, before centrifugation at 3,000 r/min for 15 min at 4 °C, and extraction of the supernatant. The contents of sIgA in each intestinal segment and HSP70 in serum were determined by ELISA kits (Enzyme immunoassay, Jiangsu, China). Glucose (GLU) levels as well as glutamic oxaloacetic transaminase (AST) and glutamic alanine transaminase (ALT) activities in serum were determined using the commercial kits (Nanjing Jiancheng Bioengineering Institute, Nanjing, China). Samples were analysed according to the manufacturer’s instructions.

### Gene expression

The expression of intestinal inflammation-related genes *TNF-α*, *IL-2* and *IL-4*, intestinal Bet transporter-related genes *SLC9A3*, *SLC7A6*, *SLC36A1*, *SLC6A20* and *SLC38A2*, and mTOR pathway-related genes *mTOR*, *P70S6k1* and *4EBP-2* was determined using real-time fluorescence quantitation (RT-PCR).

Total RNA was extracted from each intestinal tissue sample using Trizol reagent and the integrity of the extracted RNA was evaluated at an absorbance ratio of 260/280 nm (1.8–2.0 is the ideal ratio). Genomic DNA was removed using the PrimeScript RT kit before reverse transcription was carried out according to the manufacturer’s instructions. Real-time PCR analysis was performed using a SYBR^®^Primix Ex Taq^TM^ II kit (TaKaRa, Shiga, Japan) and a fluorescent quantitative PCR instrument (Applied Biosystems 7900HT, Foster city, California, USA). The relative gene expression was calculated by the 2^−ΔΔct^ method using β-action as the internal reference gene. The primer sequences are shown in Table 2 and the primer synthesis was performed at Shanghai Biotechnology Co., Ltd.

**Table 2:** Primer sequences for quantitative real-time polymerase chain reaction

**Gene**	**Primer sequences (5′→3′)**	**Product length, bp**	**Accession number**
β-action	F: CAGAAACGAGACGAGATTGGC R: CAATCAAAGTCCTCGGCCACA	189	NM_001101683.1
*IL-*2	F: TGCCCAAGAAGGTCACAGAA R: CCCCCATGAGAGTTTTTGCC	106	NM_001163180.1
*IL-*4	F: GCGACATCATCCTACCCGAA R: TCGGTTGTGTTCTTGGGGAC	115	NM_001163177.1
*mTOR*	F: CTTGAGTCCGAAGAGGATGG R: ATGAGGGTGGCAAGAAAGTG	187	XM_008262137.2
*P70S6k1*	F: TGTGACGACAAGTGGGGAAG R: TGGCCGTTTGGAGATCATGG	100	NM_001101690.1
*SLC6A20*	F: GGATGGCTGACTCAAGAACC R: CACACACTGAGGCGTAGAGC	226	XM_008269584.2
*SLC7A6*	F: TGCCCCTCTTCAGTGATACC R: GCAAAGCTTCTGGGTGACTC	161	XM_017342318.1
*SLC9A3*	F: ATCGGCTCATTGAGGTGAAC R: TCCCATTGGTGATTGGTGAT	198	NM_001082107.1
*SLC36A1*	F: ACTACTCGGAGGGCATGAGT R: TGAAGATGGGAGCGTTGCTT	135	XM_008254846.2
*SLC38A2*	F: TGTGCAGCCAAAGATTTCAG R: GACGGGAGGATGAAGATCAA	158	XM_002712695.3
*TNF-*α	F: AGCCCACGTAGTAGCAAACC R: TGAGTGAGGAGCACGTAGGA	192	XM_008262537.2
*4EBP-*2	F: GTTGGATCGTCGCAATTCTCC R: ATTAAGGTGCCAGGGCTAGTG	87	XM_002718436.3

*IL-2* Interleukin-2, *IL-4* Interleukin-4, *mTOR* Mammalian target of rapamycin, *P70S6k1* P70 ribosomal protein S6 kinase 1, *SLC7A6* Solute carrier family 7 member 6, *SLC9A3* Solute carrier family 9 member 3, *SLC6A20* Solute carrier family 6 member 20, *SLC36A1* Solute carrier family 36 member 1, *SLC38A2* Solute carrier family 38 member 2, *TNF-α* Tumor necrosis factor-alpha, *4EBP-2* Eukaryotic initiation factor 4E-binding protein-2

### Statistical analysis

The data were analysed by one-way ANOVA using SAS 9.4 (V9.4, SAS Institute Inc., Cary, NC, USA) statistical software and combined with Tukey’s method for multiple comparisons, and regression analysis was performed using SAS 9.4 software. The weighted least square method was used to analyse the mortality, morbidity and health risk index of rabbits at the unit of treatment, and the Pearson correlation coefficient between some gene expressions was obtained by correlation analysis. All data results are expressed as mean and standard error of mean (SEM), with *P* < 0.05 as a significant difference and 0.05 ≤ *P* < 0.10 as a trend.

## Results

### Effects of betaine on growth performance and health status of growing rabbits in summer heat

During the experiment, the average daily temperature of the rabbitry was 30.48 °C, the relative humidity was 69.44%, and the average THI was 29.38, indicating that the rabbits had been under severe heat stress (Table 3). Compared with the control group, the dietary addition of Bet had no significant effect on BW, ADG, ADFI and F/G in growing rabbits from 1 to 21 d (Table 4). Dietary supplementation with different levels of Bet had no significant effects on morbidity, mortality and health risk index of growing rabbits compared with the control group without Bet (Table 5).

**Table 3:** Changes of the THI of the rabbitry during the experimental period

**Items**	**Temperature, °C**		**Humidity, %**		**THI**
	**6:00**	**14:00**	**6:00**	**14:00**	
1^st^ week	27.45	35.18	71.21	54.96	29.81
2^nd^ week	26.97	33.47	81.07	66.86	29.29
3^rd^ week	26.24	33.62	81.89	60.68	29.06

*THI* Temperature humidity index

**Table 4:** Effects of dietary betaine on growth performance of growing rabbits from 1 to 21 d under the summer heat

**Items**	**Betaine, g/kg**					**SEM**	***P*-value**		
	**0**	**0.75**	**1.00**	**1.50**	**2.00**		**ANOVA**	**Linear**	**Quadratic**
1 d BW, g	749.16	748.74	750.11	750.00	745.47	4.10	0.997	0.825	0.941
21 d BW, g	1,261.37	1,260.74	1,260.21	1,284.95	1,251.26	9.00	0.822	0.967	0.892
ADG, g/d	24.39	24.38	24.29	25.47	24.09	0.36	0.780	0.869	0.903
ADFI, g/d	69.46	69.56	73.42	72.56	69.43	0.63	0.106	0.598	0.173
F/G	2.90	2.90	3.05	2.87	2.95	0.04	0.633	0.822	0.874

*BW* Body weight, *ADG* Average daily gain, *ADFI* Average daily feed intake, *F/G* Feed to gain ratio

**Table 5:** Effects of dietary betaine on health status of growing rabbits under the summer heat

**Items**	**Betaine, g/kg**					**SEM**	***P*-value**	
	**0**	**0.75**	**1.00**	**1.50**	**2.00**		**ANOVA**	**Linear**
Morbidity, %	13.33	10.00	10.00	6.67	6.67	-	0.894	0.323
Mortality, %	10.00	10.00	6.67	3.33	3.33	-	0.710	0.166
Health risk index, %	23.33	20.00	16.67	10.00	10.00	-	0.529	0.084

### Effects of betaine on serum stress indexes of growing rabbits in summer heat

Compared with the control group without Bet, the content of GLU in serum of growing rabbits was decreased by adding 1.0 g/kg Bet (*P* < 0.05, Table 6). When Bet was added to 2.0 g/kg, the content of GLU and HSP70 in serum was decreased (*P* < 0.05, Table 6). Dietary Bet tended to reduce the activity of AST in serum of growing rabbits (*P* = 0.059), but had no effect on the activity of ALT (*P* > 0.05) (Table 6).

**Table 6:** Effects of betaine on serum stress indexes of growing rabbits under the summer heat

**Items**	**Betaine, g/kg**					**SEM**	***P*-value**		
	**0**	**0.75**	**1.00**	**1.50**	**2.00**		**ANOVA**	**Linear**	**Quadratic**
ALT, U/L	19.51	15.88	18.83	16.64	16.29	1.05	0.766	0.465	0.760
AST, U/L	3.29^a^	3.09^ab^	2.11^ab^	1.97^b^	2.10^ab^	0.19	0.059	0.007	0.017
HSP70, ng/mL	74.34^a^	71.24^a^	74.18^a^	69.88^a^	62.70^b^	1.21	0.006	0.002	0.002
GLU, mmol/L	7.09^a^	6.45^abc^	6.28^bc^	5.80^c^	6.74^ab^	2.32	0.012	0.140	0.006

*ALT* Alanine aminotransferase, *AST* Aspartate aminotransferase, *HSP70* Heat shock protein 70, *GLU* Glucose

^a–c^Different superscript lowercase letters within the same row indicate significant differences (*P* < 0.05)

### Effect of betaine on intestinal morphology of growing rabbits in summer heat

As shown in Table 7, compared with the control group, supplementation of 1.0 and 2.0 g/kg Bet increased jejunal VH in growing rabbits (*P* < 0.05), and supplementation of 1.0 g/kg Bet also increased jejunal CD (*P* < 0.05). At the same time, dietary Bet tended to increase V/C in the jejunum and ileum of growing rabbits (*P* = 0.058, *P* = 0.079), but had no effect on VH and CD in the ileum. The jejunal VH and V/C and ileal V/C of growing rabbits increased linearly with increasing levels of Bet in the diet (*P* < 0.05).

**Table 7:** Effects of betaine on intestinal morphology of growing rabbits under the summer heat

**Items**	**Betaine, g/kg**					**SEM**	***P*-value**		
	**0**	**0.75**	**1.00**	**1.50**	**2.00**		**ANOVA**	**Linear**	**Quadratic**
Jejunum									
VH, μm	340^b^	350^b^	790^a^	530^ab^	700^a^	56.59	0.014	0.021	0.051
CD, μm	130^b^	260^b^	590^a^	110^b^	120^b^	56.91	0.013	0.673	0.068
V/C	2.60	1.64	2.76	4.76	5.75	0.05	0.058	0.007	0.013
Ileum									
VH, μm	350	380	410	500	460	25.37	0.360	0.052	0.150
CD, μm	100	110	100	100	100	2.56	0.804	0.461	0.767
V/C	3.35	3.47	4.00	5.07	4.53	0.23	0.079	0.011	0.040

*VH* Villus height, *CD* Crypt depth, *V/C* Villus height to crypt depth ratio

^a,b^Different superscript lowercase letters within the same row indicate significant differences (*P* < 0.05)

### Effects of betaine on intestinal sIgA levels of growing rabbits in summer heat

Compared with the control group without Bet supplementation, 0.75 and 1.0 g/kg Bet supplementation increased duodenal sIgA levels (*P* < 0.05, Table 8). The highest jejunal sIgA levels were found with the addition of 1.5 g/kg Bet, but jejunal sIgA levels were lower when Bet was added at 0.75 g/kg (*P* < 0.05, Table 8). Dietary supplementation with different levels of Bet increased the ileal sIgA level of growing rabbits (*P* < 0.05, Table 8), and the ileal sIgA level was the highest when the supplementation amount was 2.0 g/kg compared with the control group. With increasing Bet levels in the diet, the duodenal sIgA levels of growing rabbits showed a secondary increase followed by a decrease (*P* < 0.05), and ileal sIgA levels showed a linear increase (*P* < 0.05) (Table 8).

**Table 8:** Effect of betaine on the content of sIgA in intestine of growing rabbits under the summer heat

**Items**	**Betaine, g/kg**					**SEM**	***P*-value**		
	**0**	**0.75**	**1.00**	**1.50**	**2.00**		**ANOVA**	**Linear**	**Quadratic**
Duodenum, μg/mL	66.28^c^	80.45^a^	75.32^ab^	69.80^bc^	64.28^c^	1.57	< 0.001	0.156	0.002
Jejunum, μg/mL	74.30^ab^	69.25^b^	72.41^b^	81.33^a^	74.65^ab^	1.26	0.029	0.156	0.362
Ileum, μg/mL	72.46^c^	96.27^b^	89.00^b^	90.24^b^	124.79^a^	3.68	< 0.001	< 0.001	< 0.001

^a–c^Different superscript lowercase letters within the same row indicate significant differences (*P* < 0.05)

### Effect of betaine on the expression of intestinal inflammation-related genes in growing rabbits in summer heat

**Fig. 1:**
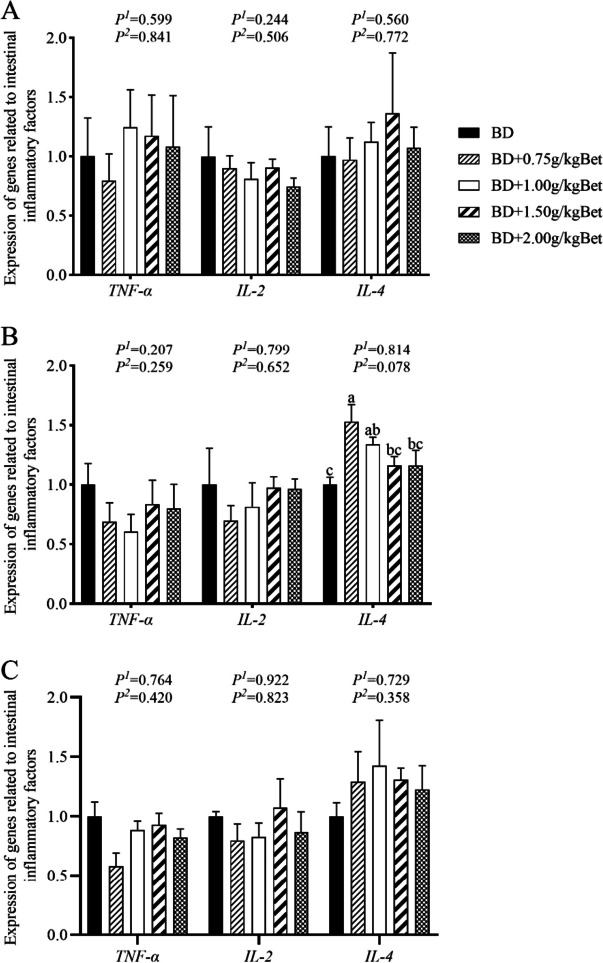
Effects of betaine on intestinal immune function of growing rabbits under the summer heat. **A** Duodenum; **B** Jejunum; **C** Ileum. *TNF-α* Tumor necrosis factor-alpha, *IL-2* Interleukin-2, *IL-4* Interleukin-4. ^a–c^ Different superscript lowercase letters indicate significant differences (*P* < 0.05). *P*^*1*^, linear; *P*^*2*^, quadratic

There was no effect of diet addition of Bet on duodenal *TNF-α*, *IL-2* and *IL-4* expression compared with the control group (Fig. 1A). Dietary Bet supplementation at 0.75 g/kg increased the expression of *IL-4* in the jejunum (*P* < 0.05), while supplementation of different levels of Bet had no effect on the expression of *TNF-α* and *IL-2* in the jejunum (Fig. 1B). Adding 0.75 g/kg Bet down-regulated the expression of *TNF-α* in the ileum (*P* < 0.05), but different levels of Bet had no effect on the expression of *IL-2* and *IL-4* in the ileum (Fig. 1C).

### Effect of betaine on the expression of genes related to betaine transporters in intestines of growing rabbits in summer heat

**Fig. 2:**
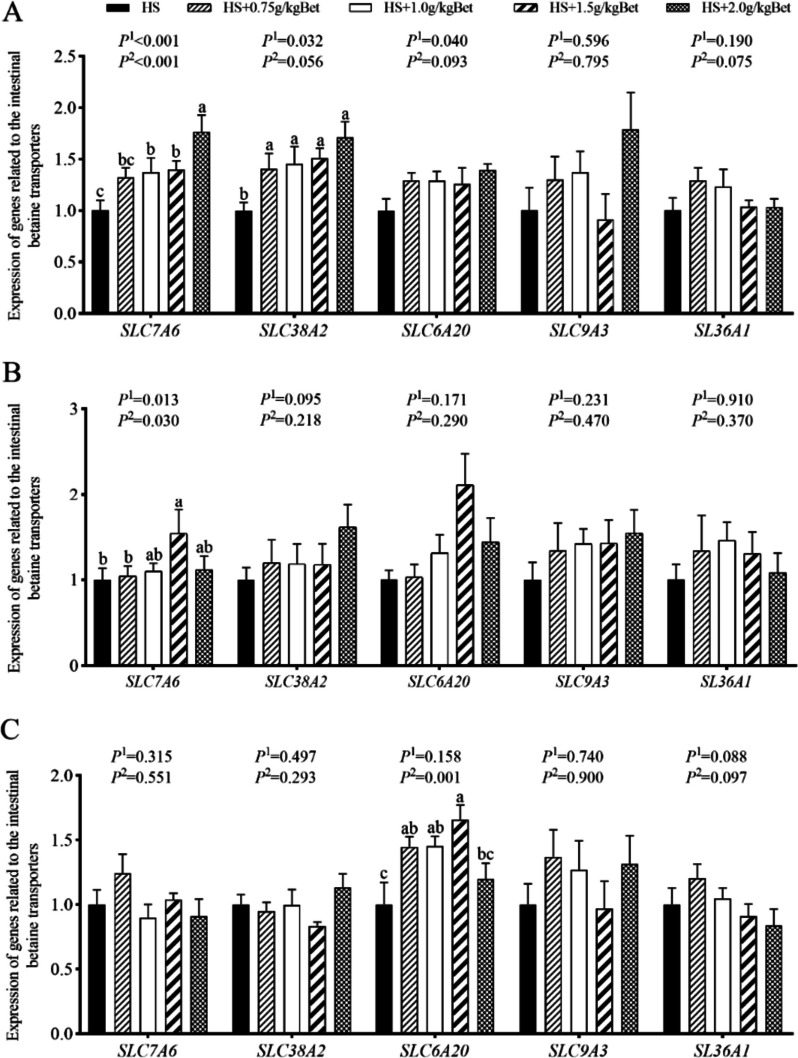
Effects of betaine on expression of betaine transporter genes in intestine of growing rabbits under the summer heat. **A** Duodenum; **B** Jejunum; **C** Ileum. *SLC7A6* Solute carrier family 7 member 6, *SLC38A2* Solute carrier family 38 member 2, *SLC6A20* Solute carrier family 6 member 20, *SLC9A3* Solute carrier family 9 member 3, *SLC36A1* Solute carrier family 36 member 1. ^a–c^ Different superscript lowercase letters indicate significant differences (*P* < 0.05). *P*^*1*^, linear; *P*^*2*^, quadratic

Compared with the group without Bet, the addition of different levels of Bet to the diet up-regulated the expression of *SLC7A6* (except for the addition of 0.75 g/kg Bet) and *SLC38A2* in the duodenum of growing rabbit (*P* < 0.05, Fig. 2A), Bet added at 1.5 g/kg up-regulated the expression of *SLC7A6* in the jejunum (*P* < 0.05, Fig. 2B), and 0.75, 1.0 and 1.5 g/kg Bet up-regulated ileal *SLC6A20* expression (*P* < 0.05, Fig. 2C). However, the addition of different levels of Bet had no effect on the expression of *SLC9A3* and *SLC36A1*, as well as duodenal *SLC6A20*, jejunal *SLC38A2* and *SLC6A20*, and ileal *SLC7A6* and *SLC38A2* in each gut segment (Fig. 2). With the increase of dietary Bet level, the expression of *SLC7A6* and *SLC38A2* in duodenum, *SLC7A6* in jejunum and *SLC6A20* in ileum of growing rabbits increased linearly (*P* < 0.05, Fig. 2).

### Effect of betaine on the expression of mTOR pathway related genes in intestines of growing rabbits in summer heat

**Fig. 3:**
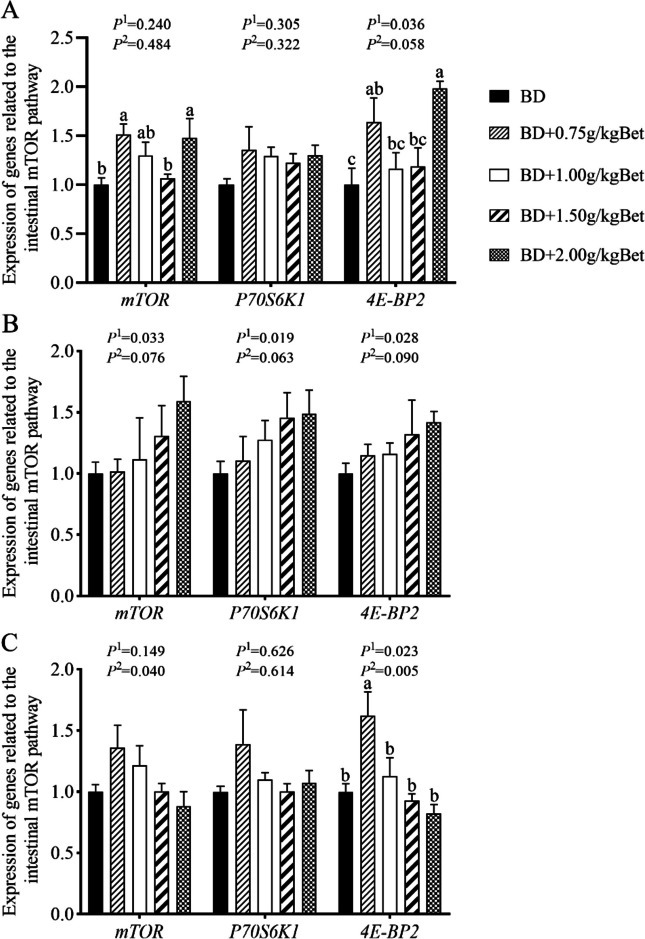
Effects of betaine on the expression of mTOR pathway genes in intestines of growing rabbits under the summer heat. **A** Duodenum; **B** Jejunum; **C** Ileum. *mTOR* Mammalian target of rapamycin, *P70S6k1* P70 ribosomal protein S6 kinase 1, *4EBP-2* Eukaryotic initiation factor 4E-binding protein-2. ^a–c^ Different superscript lowercase letters indicate significant differences (*P* < 0.05). *P*^*1*^, linear; *P*^*2*^, quadratic

Compared with the control group without Bet, diets supplemented with 0.75 and 2.0 g/kg Bet increased the gene expression of *mTOR* and *4EBP-2* in the duodenum of growing rabbits (*P* < 0.05, Fig. 3A), and diets supplemented with 0.75 g/kg Bet increased the gene expression of *4EBP-2* in the ileum (*P* < 0.05, Fig. 3C). However, dietary Bet supplementation at different levels had no effects on the gene expression of *P70S6k1* in duodenum, *mTOR*, *P70S6k1* and *4EBP-2* in jejunum, and *mTOR* and *P70S6k1* in ileum (Fig. 3). With the increase of dietary Bet level, the expression of *4EBP-2* in duodenum, *mTOR*, *P70S6k1* and *4EBP-2* in jejunum increased linearly, and the expression of *4EBP-2* in ileum decreased linearly (*P* < 0.05, Fig. 3).

### Correlation analysis of mTOR and AAT-related gene expression

As shown in Table 9, dietary Bet supplementation in growing rabbits regulated the gene expression of *mTOR* by affecting the expression of genes related to intestinal Bet transporters. There was a positive correlation between *mTOR* gene expression and *SLC7A6*, *SLC38A2*, *SLC9A3* and *SLC36A1* gene expression in duodenum of growing rabbits (*P* < 0.05), and there was a trend for a positive correlation with duodenal *SLC6A20* gene expression (*P* = 0.059). The jejunal *mTOR* gene showed a positive correlation with jejunal *SLC7A6*, *SLC38A2*, *SLC6A20* and *SLC36A1* gene expression (*P* < 0.05), and also with jejunal *SLC9A3* gene expression (*P* = 0.075). There was a positive correlation between the expression of ileal *mTOR* gene and ileal *SLC7A6*, *SLC38A2*, *SLC6A20*, *SLC9A3* and *SLC36A1* genes (*P* < 0.05).

**Table 9:** Correlation analysis of mTOR and inflammatory factor gene expression in intestines of growing rabbits

**Items**	**Duodenum**			**Jejunum**			**Ileum**		
	***TNF-α***	***IL-2***	***IL-4***	***TNF-α***	***IL-2***	***IL-4***	***TNF-α***	***IL-2***	***IL-4***
*r*	−0.096	0.042	0.302^*^	0.076	0.073	0.286	0.222	−0.10	0.354^*^
*P*	0.486	0.762	0.025	0.569	0.586	0.052	0.114	0.948	0.012

*TNF-α* Tumor necrosis factor-alpha, *IL-2* Interleukin-2, *IL-4* Interleukin-4, *r* stands for Pearson correlation coefficient, *P* stands for significance (two-tailed)

^*^Indicates significant correlation, *P* < 0.05

### Correlation analysis of mTOR and inflammation-related gene expression

Dietary Bet can regulate the expression of inflammatory factor-related genes by affecting the expression of the *mTOR* gene in the intestine of growing rabbits. There was a positive correlation between the expression of *mTOR* and *IL-4* genes in the duodenum of growing rabbits (*P* < 0.05, Table 10). The expression of the *mTOR* gene in the jejunum was positively correlated with that of the *IL-4* gene in the jejunum (*P* = 0.052), and the expression of the *mTOR* gene in ileum was positively correlated with that of the *IL-4* gene in ileum (*P* < 0.05, Table 10). However, the expression of the *mTOR* gene in duodenum, jejunum and ileum had no effect on the expression of the *TNF-α* and *IL-2* genes respectively (Table 10).

**Table 10:** Correlation analysis of mTOR and betaine transporter protein relative gene expression in intestines of growing rabbits

**Items**	**Duodenum**					**Jejunum**					**Ileum**				
	***SLC7A6***	***SLC38A2***	***SLC6A20***	***SLC9A3***	***SLC36A1***	***SLC7A6***	***SLC38A2***	***SLC6A20***	***SLC9A3***	***SLC36A1***	***SLC7A6***	***SLC38A2***	***SLC6A20***	***SLC9A3***	***SLC36A1***
*r*	0.652^**^	0.680^**^	0.258	0.329^*^	0.630^**^	0.576^**^	0.729^**^	0.572^**^	0.249	0.338^*^	0.544^**^	0.643^**^	0.345^*^	0.483^**^	0.661^**^
*P*	< 0.001	< 0.001	0.059	0.024	< 0.001	< 0.001	< 0.001	< 0.001	0.075	0.012	< 0.001	< 0.001	0.012	0.001	< 0.001

*SLC7A6* Solute carrier family 7 member 6, *SLC38A2* Solute carrier family 38 member 2, *SLC6A20* Solute carrier family 6 member 20, *SLC9A3* Solute carrier family 9 member 3, *SLC36A1* Solute carrier family 36 member 1, *r* stands for Pearson correlation coefficient, *P* stands for significance (two-tailed)

^**^Represents highly significant correlation (*P* < 0.01)

^*^Indicates significant correlation (*P* < 0.05)

## Discussion

During the summer, we observed that the THI of the rabbitry was 29.38. This value suggests that the rabbits were exposed to severe heat stress throughout the experiment. However, prolonged high temperature stimulation reduced the growth performance of rabbits, but supplementation of 1.0 or 1.5 g/kg Bet in the diet significantly improved the growth performance of rabbits [24]. Interestingly, we found that dietary betaine supplementation did not improve the growth performance of rabbits under summer heat. Experiments were performed between d 1 and 21 after weaning, which is a critical stage of rabbit gut development, so the improvement of growth performance of rabbits by Bet was not significant. Summer heat can increase morbidity and mortality, and increase the health risk index of rabbits [25]. Morbidity, mortality and health risk index are important indicators of the health status of growing rabbits [23]. It was found that HS caused 138% higher mortality in growing rabbits than in adult rabbits, indicating that HS is the most harmful to the health status of growing rabbits [25]. The results of this experiment showed that the same rate of reduction in morbidity, mortality, and health risk index (49.9%, 66.7%, and 133.3% respectively) was observed when Bet was added at 1.5 or 2.0 g/kg. This is the first time that dietary supplementation with Bet has been shown to improve the health status of growing rabbits in the summer heat to some extent. It may be because Bet as a methyl donor, can promote the conversion of excitatory amino acid Hcy to SAM, increasing the expression of inhibitory neurotransmitter GABA, inhibiting the excitability of the nervous system, reducing the respiratory rate, and enhancing the heat tolerance of rabbits, thereby reducing the occurrence of heat exhaustion [26].

Glucose is co-mediated by cellular uptake and utilisation through the glucose transporter protein family (GLUT) and the sodium-glucose cotransporter protein family (SGLT) [27]. It was found that in high temperature environments, animals can increase blood glucose level and accelerate GLU consumption in vivo by upregulating SGLT-1 and GLUT-2 expression [28, 29]. The present experimental study found that Bet reduced blood glucose levels and slowed down the energy loss from the growing rabbits under the summer heat, which is consistent with previous work [24, 30] on New Zealand white rabbits. However, these experiments found that blood glucose concentrations increased instead when the additive amount of Bet reached 2.0 g/kg. This may be due to the fact that Bet supplementation leads to an increase in serum SAM levels and promotes the production of succinate and pyruvate, precursors of gluconeogenesis, thus increasing blood glucose concentrations [31].

The levels of AST and ALT in the serum may reflect normal or abnormal liver function. It was found that summer heat causes damage to the body’s liver and a large release of AST and ALT from liver cells into the blood, leading to a significant increase in serum levels of AST and ALT [32]. The results of the present experimental study showed that Bet reduced serum levels of AST and ALT under the summer heat, which is consistent with previous studies on New Zealand white rabbits [33]. This may be due to the presence of Bet transport carriers in the liver, which promote the accumulation of Bet in hepatocytes, allowing it to exert antioxidant effects and alleviate liver damage [34]. It is shown that Bet can maintain the integrity of liver function in growing rabbits to some extent.

Prolonged exposure of animals to high temperatures enhances the expression of HSP in vivo [5], with HSP70 being the most extensive in the regulatory mechanisms of summer heat [35]. Numerous studies have found that the addition of different doses of Bet to the diets of goats [36], broilers [13], and beef cattle [37] under summer heat conditions reduced the serum levels of HSP70 to varying degrees, consistent with the results obtained in the present experiment. This may be due to the fact that Bet has a function similar to that of a heat shock protein molecular chaperone, which prevents stress-induced protein denaturation, thus stabilising protein folding and alleviating heat stress in growing rabbits to some extent [38].

VH and CD of the intestine are important for maintaining the normal morphology of the small intestine, normal nutrient absorption, and preventing bacterial translocation from the intestine [39], and small intestinal V/C reflects the developmental status of the intestine and its ability to digest and absorb nutrients [40, 41]. The results of this experiment showed that the addition of Bet to the diet increased jejunal VH and V/C and ileal V/C in growing rabbits under summer heat. These results are similar to previous studies in poultry [42] and rats [43]. Bet can maintain the stability of the intestinal morphological structure by preventing cellular water loss [44]. However, this experiment found that Bet significantly increased jejunal CD in growing rabbits under summer heat, contrary to the results of previous studies in poultry [45] and rats [43]. The increase in CD indicates poorer enterocyte maturation, possibly due to Bet promoting the production of new enterocytes, but due to the lack of data relating to the effect of Bet on the morphology of the intestine in growing rabbits under summer heat, this needs to be further investigated.

The sIgA is the main antibody of intestinal mucosal immunity and plays an important role in the clearance of pathogens and harmful substances, maintenance of intestinal environmental homeostasis and intestinal mucosal immunity. TNF-α is mainly produced by macrophages and monocytes, has pro-inflammatory properties and plays an important role in apoptosis, cell proliferation and immune response [46]. IL-4, as an anti-inflammatory factor, has a role in downregulating inflammatory response and antagonising inflammatory mediators [47]. In this study, Bet not only increased the level of sIgA in the small intestinal mucosa, but also up-regulated the gene expression of *IL-4* and down-regulated the gene expression of *TNF-α*, enhancing the immune function of the intestine. Alhotan et al. found that sIgA levels in the jejunum of broiler chickens were also significantly increased compared to the control group when supplemented with 1.0 g/kg Bet in high temperature-stressed broilers [13]. Sun et al. supplemented 3.2 g/kg Bet in grass carp and found that Bet could down-regulate the expression of the *TNF-α* gene and reduce the incidence of enteritis in the fish [48]. We also showed that Bet could improve intestinal immune function by increasing the content of intestinal immunoglobulins and regulating the expression of inflammatory factors to resist the damage caused by summer heat in growing rabbits.

The amino acid transporters SNAT2, IMINO, y+LAT2 and PAT1 that can transport Bet exist in the intestinal tract of animals [49–51], and are encoded by *SLC* gene family *SLC38A2*, *SLC6A20*, *SLC7A6* and *SLC36A1*, respectively. SLC38A2 can be used as a positive regulator of mTOR to participate in protein synthesis and amino acid uptake [18, 52]. After mTOR is activated, it can down-regulate the gene expression of pro-inflammatory factors by activating the phosphorylation state of downstream signalling factors 4EBP-2 and P70S6k1, and alleviate intestinal inflammation [53]. The results of this study indicate that dietary Bet supplementation can significantly up-regulate the gene expression of *SLC7A6*, *SLC6A20*, *SLC38A2*, *mTOR*, *4EBP-2*, and *P70S6k1* in the intestine of growing rabbits under summer heat. Correlation analysis showed that the expression of *mTOR* gene in the intestine of growing rabbits was significantly positively correlated with the expression of Bet transporters *SLC7A6*, *SLC38A2*, *SLC6A20* and *SLC36A1* genes in the intestine, and was also significantly positively correlated with the anti-inflammatory factor *IL-4* in the intestine. These results indicate that Bet can up-regulate the gene expression of tight junction proteins and anti-inflammatory factors in the intestine of growing rabbits through AAT/mTOR pathway, reduce intestinal inflammation induced by summer heat, and maintain intestinal health. This is the first time a study has confirmed this previous hypothesis [54]. However, this study found that *mTOR* gene expression had no significant effect on the gene expression of pro-inflammatory factors *TNF-α* and *IL-2*, suggesting that *mTOR* mainly plays an anti-inflammatory role by up-regulating the expression of anti-inflammatory factors.

## Conclusion

Dietary Bet supplementation in growing rabbits under summer heat improved intestinal morphology, and also up-regulate the expression of anti-inflammatory factors through the AAT/mTOR pathway, improved intestinal immune function, improved intestinal health, and alleviated the intestinal damage caused by summer heat, with the best results achieved when the amount of Bet was 2.0 g/kg. These results provide a theoretical basis for future research on the effects of dietary betaine addition on different physiological stages of rabbits.

## Data Availability

All data generated or analyzed during this study are available from the corresponding author upon reasonable request.

## Abbreviations

AAT: Amino acid transporters
ADFI: Average daily feed intake
ADG: Average daily gain
ALT: Alanine aminotransferase
AST: Aspartate aminotransferase
BD: Basal diet
Bet: Betaine
BW: Body weight
CD: Crypt depth
F/G: Feed to gain ratio
GLU: Glucose
HSP70: Heat shock protein 70
IL-2: Interleukin-2
IL-4: Interleukin-4
mTOR: Mammalian target of rapamycin
P70S6k1: P70 ribosomal protein S6 kinase 1
sIgA: Secretory immunoglobulin A
SLC7A6: Solute carrier family 7, member 6
SLC38A2: Solute carrier family 38, member 2
SLC6A20: Solute carrier family 6, member 20
SLC9A3: Solute carrier family 9, member 3
SLC36A1: Solute carrier family 36, member 1
THI: Temperature humidity index
TNF-α: Tumor necrosis factor-alpha
VH: Villus height
V/C: Villus height to crypt depth ratio
4EBP-2: Eukaryotic initiation factor 4E-binding protein-2
